# Whole-Transcriptome Analysis in Peripheral Blood Mononuclear Cells from Patients with Lipid-Specific Oligoclonal IgM Band Characterization Reveals Two Circular RNAs and Two Linear RNAs as Biomarkers of Highly Active Disease

**DOI:** 10.3390/biomedicines8120540

**Published:** 2020-11-26

**Authors:** Leire Iparraguirre, Danel Olaverri, Telmo Blasco, Lucía Sepúlveda, Tamara Castillo-Triviño, Mercedes Espiño, Lucienne Costa-Frossard, Álvaro Prada, Luisa María Villar, David Otaegui, Maider Muñoz-Culla

**Affiliations:** 1 Multiple Sclerosis Group, Neurosciences Area, Biodonostia Health Research Institute, 20014 San Sebastian, Spain; leire.iparraguirre@biodonostia.org (L.I.); a904612@alumni.unav.es (D.O.); tblasco@tecnun.es (T.B.); lucia.sepulveda@biodonostia.org (L.S.); david.otaegui@biodonostia.org (D.O.); 2 Department of Biomedical Engineering and Sciences, Tecnun-Universidad de Navarra, Manuel de Lardizábal 15, 20018 San Sebastián, Spain; 3 Spanish Network of Multiple Sclerosis, 08028 Barcelona, Spain; luisamaria.villar@salud.madrid.org; 4 Multiple Sclerosis Group, Neurosciences Area, Biodonostia Health Research Institute, Neurology Department, Basque Health Service, 20014 San Sebastian, Spain; TAMARA.CASTILLOTRIVINO@osakidetza.eus; 5 Departments of Immunology and Neurology, Multiple Sclerosis Unit, Hospital Ramon y Cajal, (IRYCIS), 28034 Madrid, Spain; mercedes.espino@salud.madrid.org (M.E.); lucienne.costa@salud.madrid.org (L.C.-F.); 6 Multiple Sclerosis Group, Neurosciences Area, Biodonostia Health Research Institute, Immunology Department, Basque Health Service, 20014 San Sebastian, Spain; ALVAROJOSE.PRADAINURRATEGUI@osakidetza.eus

**Keywords:** multiple sclerosis, biomarkers, transcriptome, microRNAs, circular RNAs, oligoclonal bands

## Abstract

The presence of anti-myelin lipid-specific oligoclonal IgM bands (LS-OCMBs) has been defined as an accurate predictor of an aggressive evolution of multiple sclerosis. However, the detection of this biomarker is performed in cerebrospinal fluid, a quite invasive liquid biopsy. In the present study we aimed at studying the expression profile of miRNA, snoRNA, circRNA and linearRNA in peripheral blood mononuclear cells (PBMCs) from patients with lipid-specific oligoclonal IgM band characterization. We included a total of 89 MS patients, 47 with negative LS-OCMB status and 42 with positive status. Microarray (miRNA and snoRNA) and RNA-seq (circular and linear RNAs) were used to perform the profiling study in the discovery cohort and candidates were validated by RT-qPCR in the whole cohort. The biomarker potential of the candidates was evaluated by ROC curve analysis. RNA-seq and RT-qPCR validation revealed that two circular (hsa_circ_0000478 and hsa_circ_0116639) and two linear RNAs (*IRF5* and *MTRNR2L8*) are downregulated in PBMCs from patients with positive LS-OCMBs. Finally, those RNAs show a performance of a 70% accuracy in some of the combinations. The expression of hsa_circ_0000478, hsa_circ_0116639, *IRF5* and *MTRNR2L8* might serve as minimally invasive biomarkers of highly active disease.

## 1. Introduction

Multiple sclerosis (MS) is a chronic, inflammatory, neurodegenerative and demyelinating disease of the central nervous system (CNS). It is considered an autoimmune disease, in which peripheral autoreactive lymphocytes enter the CNS and develop an immune response, leading to demyelinating lesions, both in white and grey matter [1]. It is estimated that about 2–3 million people have MS and it typically affects young adults, with an onset between 20 and 40 years of age. Moreover, MS is three times more prevalent in women than men and there is evidence that this ratio has increased in the last 70 years [1], a phenomenon shared with several other autoimmune diseases.

Both the disease course and clinical phenotype of MS are highly variable among patients and also with time in the same individual. For this reason, biomarkers can aid in the diagnosis, the differentiation of MS phenotypes and also in the monitoring of disease progression [2,3].

Disease activity biomarkers can be associated with the different pathophysiological processes of the disease and they could ideally help to distinguish between patients with an aggressive course and those with a more benign form [2]. In this context, the presence restricted to cerebrospinal fluid (CSF) of anti-myelin lipid-specific oligoclonal IgM bands (LS-OCMBs) has been defined as an accurate predictor of an aggressive evolution [4]. However, for this test, cerebrospinal fluid sample is needed, a quite invasive liquid biopsy. Therefore, a great effort has been done towards the discovery of minimally invasive biomarkers such as blood biomarkers [5].

In this context, gene expression profiling studies have been widely used for new biomarker discovery, but also to elucidate the molecular mechanisms underlying the pathogenic processes occurring in the disease [6,7]. In addition to the classical protein-coding transcriptome, non-coding transcriptome has gained great interest in the last decades, and several authors, including our group, have demonstrated that it has a role in MS pathogenesis [8,9,10]. MicroRNAs are the best studied class of small non-coding RNAs, although others such as small nucleolar RNAs have also been related to MS [11,12]. MiRNAs are single-strand small non-coding RNAs that regulate gene expression at a post-transcriptional level, binding to their target mRNAs, and they participate in almost all known biological processes [13]. More recently, circular RNAs (circRNAs) have emerged as new players in the RNA field, with an important role in the post-transcriptional regulatory mechanisms. They have been found to participate in several processes such as tumor, metabolism and immune-related pathways and consequently, they have also been linked to various diseases such as neurological and autoimmune diseases including a few studies in MS [14,15,16,17,18]. Both miRNA and circRNA have been proposed as promising source of biomarkers in a wide variety of sample types, such as blood, serum, saliva, urine and CSF [19,20,21].

In light of these evidences, and due to the increasing need for a solid, reproducible and accessible biomarker for MS, we hypothesized that a whole-transcriptome study of peripheral blood mononuclear cells (PBMCs) from patients with LS-OCMBs characterization could reveal new biomarkers that correlate with this stablished CSF marker. Such a biomarker could be used more easily to monitor patients on an ongoing basis, since blood sampling is less invasive, with less adverse effects, than doing a lumbar puncture.

With this in mind, in the present study we analyzed the expression of small non-coding RNA, circRNA and linear RNAs in peripheral blood mononuclear cells (PBMCs), from 89 patients with positive (*n* = 42) and negative (*n* = 47) LS-OCMBs characterization, and found two circular RNAs and two linear RNAs, with differential expression between the two groups, that could serve as future blood biomarkers of highly active disease.

## 2. Experimental Section

### 2.1. Patients, Sample Collection and RNA Isolation

MS patients were recruited in the Hospital Ramon y Cajal after giving informed consent. Blood samples were obtained and PBMCs were isolated following a standard Ficol gradient separation protocol and frozen in liquid nitrogen until used. Total RNA was isolated using the miRNeasy mini kit (Qiagen, Hilden, Germany) following the manufacturer’s instructions. RNA concentration was measured using the NanoDrop ND-1000 spectrophotometer (ThermoFisher Scientific, Waltham, Massachusetts, MA, USA), and the que quality of the samples included in microarray and RNA-seq experiments was assessed using the Agilent 2100 Bioanalyzer (Agilent Technologies, Santa Clara, CA, USA), obtaining a RNA integrity number higher than 6 in all the samples. CSF was obtained to analyze the status of LS-OCMBs as previously described [4,22].

The main clinical and demographical characteristics of patients are summarized in Table 1. A complete list of samples and the experiment in which each of them was included is available in Table S1. The study was approved by the hospital’s ethics committee (MMC-UEM-2018-01, October 2018). The profiling cohort includes only female samples, aiming at reducing the source of variability and considering that MS is three times more prevalent in females than men in this disease [1].

### 2.2. Microarray Analysis

Total RNA (200 ng) was labeled using the FlashTag HSR Biotin labelling kit (Genisphere LLC, Hatfield, Pennsylvania, PA, USA) and hybridized to the GeneChip miRNA 4.0 Array (Affymetrix, Santa Clara, CA, USA), which covers 2578, 2025 and 1996 human mature miRNAs, pre-miRNAs and snoRNAs, respectively. Labeled RNA was hybridized to the array, washed and stained in a GeneChip Fluidics Station 450 and scanned in a GeneChip Scanner 7G (Affymetrix, Santa Clara, CA, USA).

Microarray data analysis was carried out in Transcriptome Analysis Console 4.0 software (Affymetrix, Santa Clara, CA, USA), applying the RMA + DABG algorithm only to human probesets for normalization, detection and summarization purposes. We performed a classical differential expression analysis between patients with positive and negative LS-OCMBs, considering as differentially expressed those probesets showing a *p*-value lower than 0.01 and an absolute fold-change (FC) value higher or equal to 1.5.

### 2.3. RNAseq

Library preparation and Next Generation Sequencing was performed at CD Genomics (Shirley, New York, NY, USA). The concentration and quality of the RNA samples was again measured using Agilent 2100 Bioanalyzer before library preparation. After normalization, rRNA was depleted from the total RNA sample using the Ribo-Zero rRNA removal kit and followed by purification and fragmentation steps. To construct the sequencing libraries, a strand-specific cDNA synthesis was performed, the 3′ ends were adenylated and adaptors were ligated. The resulting libraries were subjected to a quality control and normalization process. Paired-end sequencing was performed with Illumina HiSeq X Ten PE150 (Illumina, San Diego, CA, USA).

The data presented in this study (raw data of microarray and RNA-seq experiments) are openly available in Gene Expression Omnibus (GEO) under the series reference GSE159036.

### 2.4. CircRNA Detection and Quantification in RNA-Seq Data

First, quality of the sequencing was checked and, then, reads were mapped to the human genome (hg19, downloaded from UCSC Genome Browser [23]) using STAR version 2.5.4b (https://code.google.com/archive/p/rna-star/) [24] or BWA version 0.7.17-1 (http://maq.sourceforge.net) [25]. Subsequently, circRNA prediction was performed by CIRCexplorer2 version 2.3.3 (https://circexplorer2.readthedocs.io/en/latest/) [26] and CIRI2 version 2.0.5 (https://sourceforge.net/projects/ciri/) [27] adhering to the recommendation by the authors. Moreover, only circRNAs supported by both algorithms were considered as bona fide circRNAs and used in subsequent analyses, a method that has been described in the literature by other authors [28]. CircRNA expression was based on back-spliced junctions-spanning reads according to CIRI2 quantification. After filtering out circRNAs with low expression (sum of reads < 10), differential expression analysis was performed using DESeq2 version 1.28.1 (http://www.bioconductor.org/packages/release/bioc/html/DESeq2.html) [29] package for R (version 3.6.3) (https://www.r-project.org/) in R-studio (Version 1.0.136) (https://rstudio.com/), considering as differentially expressed those circRNA showing a *p*-value < 0.05 and a fold-change higher than 2. To select a group of candidates for validation purposes, two additional filters were applied: (1) BaseMean value higher than five (BM > 5) and (2) the transcripts having a read count value of zero in any of the samples were removed. Finally, we selected ten candidate circRNAs having the highest absolute fold-change value.

### 2.5. Linear RNA Detection and Quantification in RNA-Seq Data

After sequencing and quality control, the Kallisto version 0.44.0 (http://pachterlab.github.io/kallisto/) [30] algorithm was used for transcript pseudoalignment, identification and quantification. Differential expression analysis was performed using DESeq2 package. Before running the DESeq2 algorithm, we applied a filter of a minimum number of reads (sum of number of reads higher than or equal to 10) to remove low expression transcripts. For subsequent analysis, due to the large number of transcripts detected and our small sample size, we added a filter to keep only the most robust transcripts regarding their read count. Therefore, we created a detection filter, as follows: (1) Transcripts detected in both groups—transcripts having at least two reads in ¾ of samples from each group. (2) Transcripts detected only in one of the groups—transcripts having at least two reads in ¾ of samples in the negative group but only in 1 sample from the positive group (detected only in negative group), and transcripts having at least two reads in ¾ of positive group but only in 1 from negative group (detected only in positive group). Transcripts selected with this detection filter were considered as consistent linear RNAs. Finally, differentially expressed transcripts were considered those showing a *p*-value < 0.05 and an absolute fold-change value higher than two. For validation purposes, we selected ten candidate linear RNAs having the highest absolute fold-change value and having an assigned gene name.

**Figure 1:**
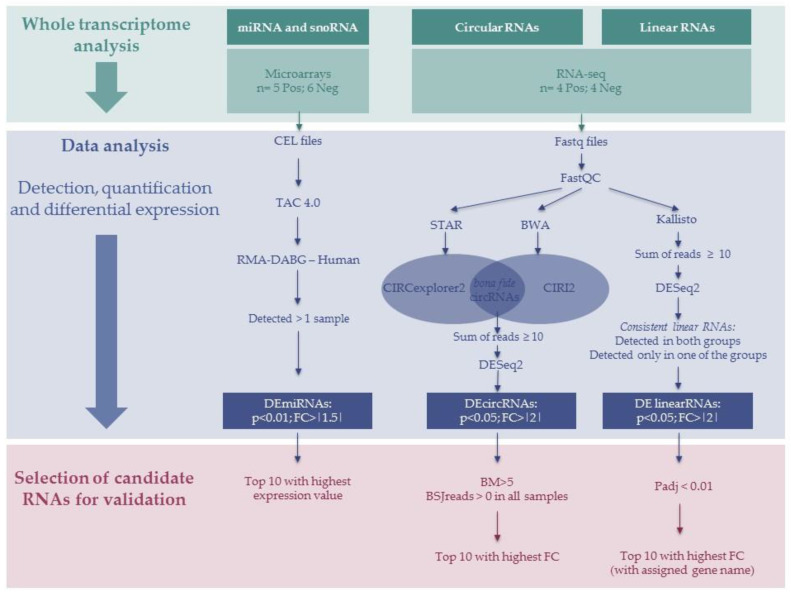
Summary of the study indicating profiling experiments, data analysis tools, filters and candidate selection criteria. Pos—positive. Neg—negative. DE—differentially expressed. FC—fold-change. BM—BaseMean. BSJ—backspliced junction. Padj—adjusted *p*-value. BWA—Burrows-Wheeler Aligner.

All these profiling experiments and data analysis steps, tools and filters are summarized in Figure 1.

### 2.6. cDNA Synthesis and Quantitative PCR

For the validation of candidate microRNAs, total RNA (10 ng) was reverse transcribed into cDNA using TaqMan^TM^ Advanced miRNA cDNA synthesis kit (Applied Biosystems^TM^, Foster City, CA, USA). To quantify miRNA expression, we used TaqMan Advanced miRNA assays and TaqMan Fast Advanced Master Mix (Applied Biosystems^TM^, Foster City, CA, USA), following the manufacturer’s protocol. Assay for hsa-miR-191-5p was used as a reference miRNA for normalization purposes.

For the validation of candidate circRNA, total RNA (500 ng) was reverse transcribed into cDNA with random primers using the High Capacity cDNA Reverse Transcription Kit (Applied Biosystems, Foster City, CA, USA), following the kit protocol. PCR reaction was set up using 10 ng of cDNA as the template and using Power SYBRGreen Master Mix (Applied Biosystems^TM^ Foster City, CA, USA) with the following thermal cycling program: A temperature of 50 °C for 2 min, 95 °C for 10 min, 40 cycles of 95 °C for 15 s and 60 °C for 1 min, followed by a dissociation curve analysis. Divergent primers were used so that the amplicon spans the backspliced junction (BSJ), as described previously [18]. EEF1A1 and B2M were used as the reference genes, using the mean value of both genes for normalization purposes. The presence of a single-peak in the melting curve indicated the specificity of the amplification.

For the validation of candidate linear RNAs, total RNA (500 ng) was reverse transcribed into cDNA with oligo-dT and random primers using the miScript II RT (Qiagen, Hilden, Germany) kit with the HiFlex buffer, as indicated in manufacturer’s protocol. PCR reaction was set up using 10 ng of cDNA as template and using miSscript SYBRGreen PCR kit (Qiagen, Hilden, Germany) with the following thermal cycling program: A temperature of 95 °C for 15 min, 40 cycles of 94 °C for 15 s, 55 °C for 30 s and 70 °C for 30 s, followed by a dissociation curve analysis. The amplification of each linear RNA was carried out with QuantiTect primer Assays (Qiagen, Hilden, Germany) (Table S2) and B2M was used as a reference gene for normalization. The presence of a single-peak in the melting curve indicated the specificity of the amplification.

All retrotranscription reactions were run in a Veriti Thermal Cycler (Applied Biosytems, Foster City, CA, USA) and quantitative PCR reactions were performed in a CFX384 Touch Real-Time PCR Detection System (Bio-Rad laboratories, Inc., Hercules, CA, USA).

Prior to run all the validation experiments, a technical validation of the circRNA amplification was carried out. The RT-qPCR amplification products were subsequently purified with the ExoSAP-IT™ PCR Product Cleanup Reagent (Applied Biosystems, Foster City, CA, USA) following the manufacturer’s instructions and Sanger sequenced (ABIprism 3130) (Applied Biosystems, Foster City, CA, USA) in order to check for the presence of the BSJ. In addition, the PCR products were also subjected to electrophoresis on agarose gel to confirm the presence of a single amplification product.

The Raw Cq values and melting curves were analyzed in CFX Maestro 1.0 (Bio-Rad laboratories, Inc., Hercules, CA, USA). Expression levels represented as fold-change (FC) were calculated using the 2^DDCq method. Statistical analysis was done by R in RStudio on DCq data. Distribution of data was tested with Shapiro–Wilk test and the difference of the distribution was assessed by Student t-test or non-parametric Wilcoxon Rank sum test. In each circRNA and linear RNA dataset, outlier samples were removed before the statistical analysis, identifying those values using boxplot.stat function in R.

### 2.7. Gene Overrepresentation Test

In order to describe the function of differentially expressed transcripts, the PANTHER overrepresentation test was applied (released 7 November 2019, Panther [31]), based on the Complete Biological Process from Gene Ontology database (released 9 December 2019). As a reference background, we used the list of genes giving rise to detected transcripts defined above. Fisher test was carried out and false discovery rate (FDR) correction was applied to raw *p*-values, taking as a significant threshold FDR values below 0.05.

### 2.8. ROC Curve Analysis

Based on DCq values obtained by RT-qPCR, we performed a receiver operating characteristic curve (ROC curve) analysis in R environment in RStudio. Three different datasets were used: (1) circRNA validation data, (2) linear RNA validation data and (3) samples in both datasets to test different combination of transcripts. This last dataset was smaller given that we needed to include only samples without any missing value (Table S1). The combination of different transcripts was computed with ROC function of “Epi” package.

## 3. Results

### 3.1. microRNA Expression Profile

**Figure 2:**
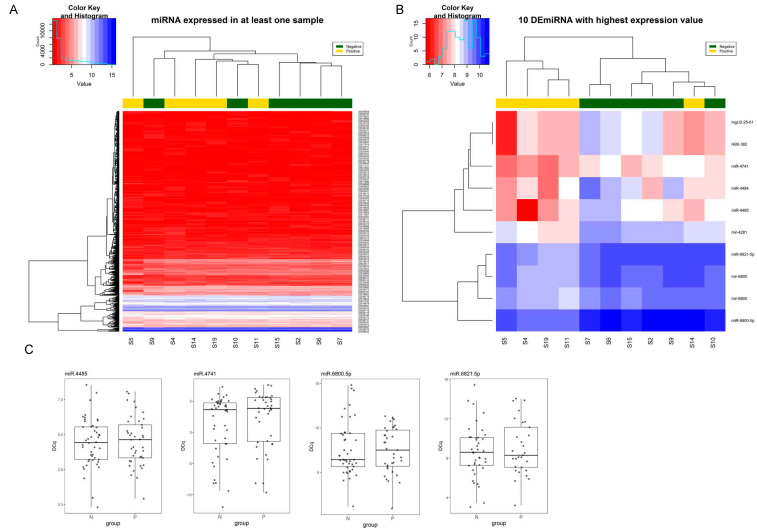
Results of miRNA expression profile by microarrays. (**A**) Heatmap showing the expression (microarray intensity signal) of miRNA that are expressed in at least one sample. (**B**) Heatmap of the expression of the ten DEmiRNAs showing the highest expression. Hierarchical clustering shows that the expression pattern of these ten miRNAs is able to group patients according to their LS-OCMBs status. (**C**) RT-qPCR validation results of selected candidate miRNAs. P—positive LS-OCMB status. N—negative LS-OCMB status. DE—differentially expressed.

Microarray analysis was able to detect 2827 probesets in at least one sample out of the total 6659 human probesets (42.45%) (Figure 2A). Among detected probesets, 33 showed a differential expression between positive and negative patients for LS-OCMBs, and more importantly, the top 10 probesets that show the highest expression are able to group patients according to their LS-OCMBs status (Figure 2B). Among these 10 probesets, we can find five mature miRNA, two pre-miRNA and two small nucleolar RNAs. For validation purposes, we selected the four mature miRNA for which TaqMan Advanced miRNA Assays were available (hsa-miR-6800-5p, hsa-miR-6821-5p, hsa-miR-4485 and hsa-miR-4741). However, RT-qPCR validation results did not confirm the alteration of these four miRNAs (Figure 2C).

### 3.2. circRNA Expression Profile

**Figure 3:**
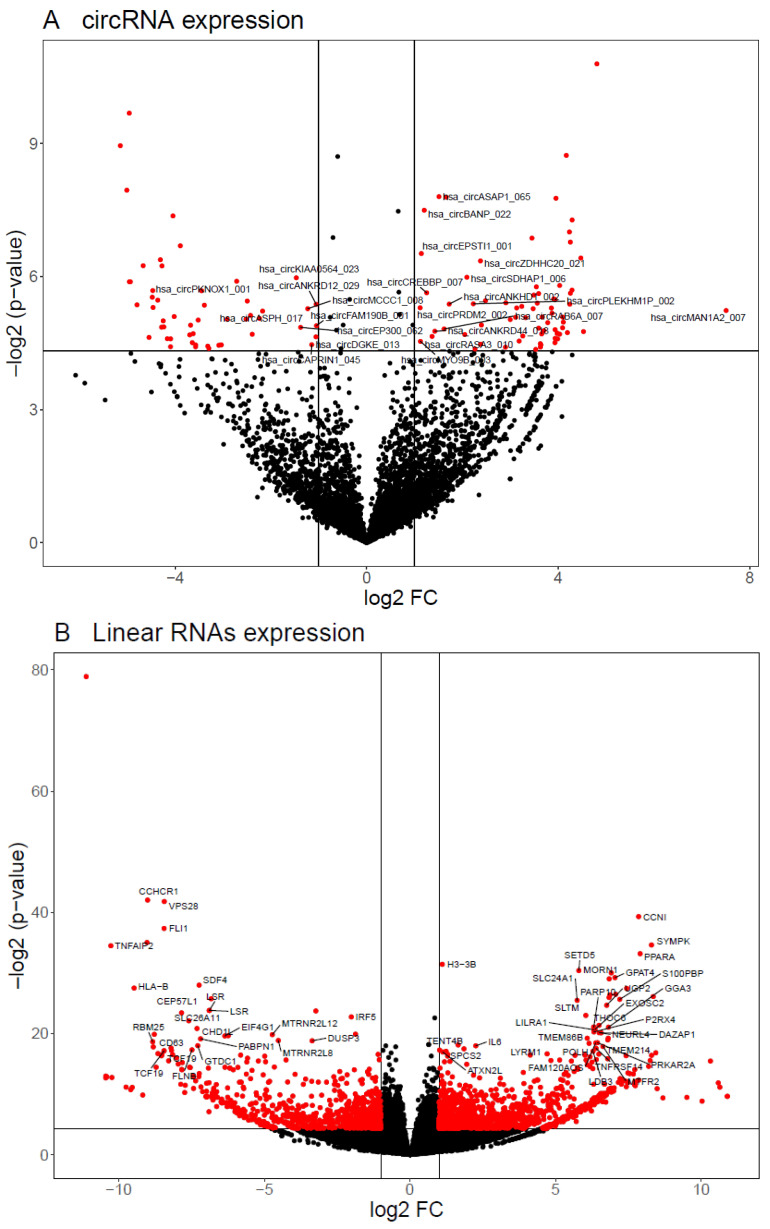

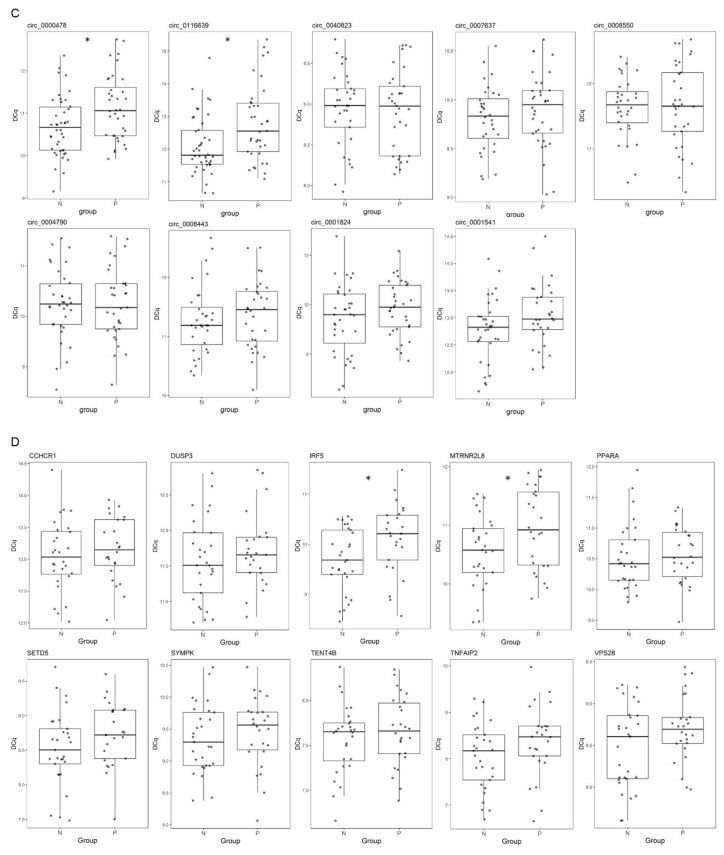
Results of circRNAs and linear RNAs expression profile by RNA-seq. (**A**) Volcano plot showing the expression difference (log2 FC) between positive and negative group of circRNA that show a sum of reads across all samples higher than 10. DEcircRNA are highlighted in red and labels point at those DEcircRNA with a base mean higher than 5. (**B**) Volcano plot showing the expression difference (log2 FC) of linear RNAs between positive and negative groups. Differentially expressed linear RNAs are highlighted in red and labels point at those DE linear RNAs with an adjusted *p*-value < 0.01. (**C**) RT-qPCR validation results of selected candidate circRNAs. Asterisk indicates a statistically significant difference (*p* < 0.01). circ_0000478 and circ_0116639 validation includes more samples than the rest of the circRNAs because of limitations in sample amount (Table S1). (**D**) RT-qPCR validation results of selected candidate linear RNAs. Asterisk indicates a statistically significant difference (*p* < 0.05). P: positive LS-OCMB status. N: negative LS-OCMB status.

RNA-seq analysis pipeline of circular transcriptome detected 27,630 bona fide circRNAs and 5431 circRNAs (19.6%) with sum of reads ≥ 10 (Figure 3A). Differential expression analysis revealed that 124 circRNAs are altered (*p* < 0.05; FC > |2|) between the two groups, 72 of them being upregulated, while 52 are downregulated.

We selected ten candidate circRNAs to confirm their differential expression by RT-qPCR in a larger cohort. First, the technical test for primer amplification confirmed a single and specific circRNA amplification for nine out of the 10 circRNA candidates. circMETRNL was the one that did not show a good amplification and a single band in agarose gel, so we discarded it in subsequent validation experiments. Additionally, Sanger sequencing of the PCR product confirmed that the amplicons span the BSJ (Figure S1). As it can be observed in Figure 3C, the lower expression of hsa_circ_0000478 and hsa_circ_0116639 was confirmed by qPCR in PBMCs from patients with positive LS-OCMBs (FC = −1.5 and FC = −1.65, respectively; *p* < 0.01).

### 3.3. Linear Transcripts Expression Profile

In the present study, we also analyzed the linear transcriptome by RNA-seq, identifying 115,869 transcripts with ≥10 reads in total. We applied an additional filter using a detection criterion, which permitted to identify 84,863 linear RNAs with a consistent expression pattern, from which 92.8% were detected in both groups. Among them, 2441 transcripts were differentially expressed (*p* < 0.05 and FC > |2|), from which 1421 were detected in both groups (Figure 3B). The rest of the transcripts are specific to one of the groups, 382 being expressed only in PBMCs from patients with negative LS-OCMBs status while 638 transcripts are only expressed in patients with positive LS-OCMBs.

We selected ten candidate linear RNAs to confirm their differential expression by RT-qPCR in a larger cohort. As it can be observed in Figure 3D, the lower expression of IRF5 and MTRNR2L8 was confirmed in PBMCs from patients with positive LS-OCMBs (FC = −1.33 in both transcripts; *p* < 0.05).

**Figure 4:**
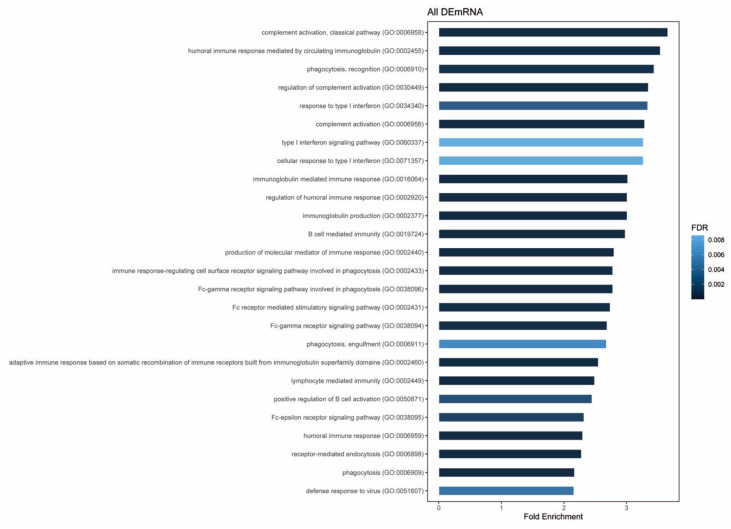
Results from gene overrepresentation test of 2441 DEmRNA. The most significant (fold-enrichment > 2) and enriched (FDR < 0.01) GO biological processes are shown. Bars are colored according to their FDR value. DE—differentially expressed. FDR—false discovery rate.

Differentially expressed linear RNAs are enriched in biological processes regarding mainly the immune system. The most significant and enriched terms (FDR < 0.01 and fold-enrichment > 2) are shown in Figure 4. Complement activation, humoral immune response and type I interferon signaling pathway appear among the top enriched terms.

### 3.4. Evaluation of circRNA and Linear RNAs as Biomarkers of a Highly Active Disease

In order to assess the potential of the four validated RNAs as blood biomarkers of the LS-OCMB status we performed the ROC curve analysis. As shown in Table 2, different combinations of RNAs were tested to find the best performance in discriminating both groups. We found that different combinations of both circRNAs and linear RNAs improves the performance, reaching AUC values of around 70%.

## 4. Discussion

In this work, we characterized the whole-transcriptome of PBMCs from MS patients with distinct LS-OCMB status. This profiling and the validation experiments revealed that the global transcriptome of PBMCs from patients with positive LS-OCMBs is different from those patients with negative LS-OCMB status.

RNA-seq results reveal that there are 124 circRNAs and 2441 linear RNAs differentially expressed between the two groups. Interestingly, 58% of linear RNAs are detected only in one of the groups, highlighting that there is a specific expression pattern in PBMCs from patients with different LS-OCMB status. It is true; however, that our RNA-seq sample size is limited and these observations should be taken with care.

To the best of our knowledge, none of the circRNAs have been previously related with MS. Circ_0000478 is located in chromosome 13 and its host gene is von Willebrand factor A domain containing 8 (*VWA8*). Interestingly, the linear transcript of this gene is also among the differentially expressed linear RNAs in our dataset, having a fold-change of −3.23 (*p* = 0.047). This transcript has been recently described and codes a mitochondrial protein with still uncertain function [32]. Regarding circ_0116639, it is located in chromosome 22, overlapping the *EP300* gene. EP300 is a histone acetyltransferase, which is detected in our study but is not differentially expressed. These differences in expression patterns of the circRNA with their host genes reveals that the biogenesis of circRNAs is independently regulated from that of their host genes, as it has been previously described [33].

Of note, interferon regulatory factor 5 (*IRF5*) is one of the confirmed downregulated transcripts, and polymorphisms in this gene has been associated to the risk of developing MS in last genome-wide association analysis, performed by the International Multiple Sclerosis Genetics Consortium, as well as in replication studies in Spanish cohorts [34,35]. Additionally, SNPs in this gene have been related to increased levels of CXCL13 in CSF, a chemokine related to highly active disease [36]. On top of that, it has been shown in animal models that IRF5 is related with the microglial polarization towards a pro-inflammatory state (M1) in response to stroke and in Alzheimer’s disease [37,38]. This could suggest microglial implication in a more aggressive disease course, as it has been suggested by other authors in a exome sequencing project [39]. Nonetheless, our findings are made in peripheral PBMCs, so the *IRF5* source might be peripheral monocytes or macrophages. In any case, further research is needed to uncover *IRF5* implication in a highly active MS disease course.

On the other hand, MT-RNR2 like 8 transcript (*MTRNR2L8*), the other validated linear transcript showing lower expression in the LS-OCMB+ group, is coded in chromosome 11, and, interestingly, it spans the location of the miR-4485 stem-loop location, one of the four miRNAs selected for validation in the present study. According to microarray data, miR-4485 is downregulated in LS-OCMB+ groups, as it is *MTRNR2L8*. The fact that *MTRNR2L8* is a host gene for mir-4485 may explain that both transcripts appear downregulated in this group of patients, even if the miRNA could not have been validated. These results point at a possible interaction between these two RNAs that might be related to a more active disease, but further research is needed to confirm this hypothesis. According to UniProt, MTRNR2L8 has a role as a neuroprotective and antiapoptotic factor and it has been found to be increased in a specific area of the brain from patients with major depressive disorder [40]. Although the function of this gene is still unclear, the possible role as a neuroprotective factor is very interesting in the case of MS, since we find this gene downregulated in PBMCs. But again, further studies should be conducted to uncover the possible link between *MTRNR2L8* expression in peripheral blood and disease activity, as well as the mechanisms underlying this relationship.

Gene overrepresentation test shows that biological processes related to complement activation, humoral immune response and type I interferon response are among the most enriched term. Of note, intrathecal synthesis of IgM antibodies has previously been correlated with intrathecal complement activation [41], and its role in demyelination and axonal damage has been clearly demonstrated [42]. Interestingly, different components of the complement system have been related to MS and plasma levels of some of the components have been proposed as MS disease state biomarkers [43]. Of note, these processes are enriched among altered genes in PBMC from patients with a marker of a high activity disease. This analysis may reveal that those patients may have an altered immune response that may explain their higher disease activity, but further and other kind of studies will be needed to explore this hypothesis.

The main aim of this study was to identify in blood a biomarker or a group of them that could differentiate between positive and negative LS-OCMB patients, thus could be used as a more accessible marker of highly active disease. ROC curve analysis revealed that these markers in combination have around 70% of accuracy, which is considered, in general, an acceptable performance [44]. Taking into account that these are measured in blood, they may help in the clinics to perform repeated measurements, while taking serial samples of CSF has serious drawbacks. Efforts are being made to define patients with a highly active disease course, given that their effective therapeutic window might be narrower than a more benign or less active disease form [45]. In line with this, several biomarkers have been proposed to identify individuals with aggressive disease course, such as CSF levels of neurofilament light chain, CXCL13 or CHI3L1 [46,47,48]. Other authors have also reported differences in non-coding transcriptome between patients with different disease activity. Quintana and colleagues studied the expression in CSF of a panel of miRNAs patients with MS with LS-OCMB characterization and patients with other neurological diseases (ONDs) [49]. They found differences in the expression of miR-30a-5p, miR-150, miR-645 and miR-191 between the OND group and the LS-OCMB+ group, but they could not find any difference between patients with positive and negative LS-OCMBs. Due to differences in the profiling platforms between their study and the present one, we are unable to check the expression of our candidate miRNA in data from Quintana and colleagues. Another study reported that serum levels of miR-24-3p correlate with disease progression index, calculated dividing EDSS value by disease duration, but the status of LS-OCMB is not measured [50]. More recently, the expression in blood of long non-coding RNAs has also been described to show the capacity of discriminating highly active MS patients from others with less active disease course, based on a age-related MS severity score [51].

All these studies highlight the need of biomarkers that are able to identify patients with highly active disease and the effort that the scientific community is doing in this direction. In addition, it is also important to have a consensus on the definition of what is an aggressive disease course, given that each study uses different parameters to measure and classify patients in each of the groups, which makes the comparison between them really challenging [52,53]. An early recognition of a patient in this group could benefit from different therapeutic advice and; therefore, the possibility to improve the long-term outcome of the disease.

Our miRNA profiling study discovered some miRNA differentially expressed between the two groups, although they have not been validated by RT-qPCR experiments. In this regard, other techniques such as droplet digital PCR (ddPCR) may help in reducing variability and discover small differences between groups. Although it has been reported that the sensitivity is not increased with ddPCR in gene expression studies, and that the performance is similar provided that the amount of starting material is enough and there are no contaminants in the reaction [54,55]. Nonetheless, it is a technique that could be considered in future validation projects aiming at definitely discarding or including those miRNAs as biomarkers for a more aggressive MS course.

## 5. Conclusions

In summary, we have identified two circRNAs and two linear RNAs which are downregulated in patients with positive LS-OCMB status. Our findings are important in the field of biomarkers for multiple sclerosis, given that they could contribute to the identification of patients with highly active disease. Additionally, an important advantage compared to other biomarkers is that they are detected in blood, allowing serial tests to monitor their levels, while this task is not so recommended for lumbar puncture. Future studies in larger cohorts will be needed to confirm their utility, but we consider that they may serve as a first screening tool to decide which patients should go into a lumbar puncture to confirm their LS-OCMB status.

## Figures and Tables

**Table 1:** Summary of patients’ clinical and demographical data.

	Microarrays			RNAseq					
Profiling cohort	LS-OCMB status	Sex	Age	LS-OCMB status	Sex	Age			
	P = 5	F = 5	35 (23–58)	P = 4	F = 4	38 (23–58)			
		M = 0			M = 0				
	N = 6	F = 6	34.5 (27–53)	N = 4	F = 4	35 (27–44)			
		M = 0			M = 0				
Validation cohort	miRNA validation *			circRNA validation *			linear RNA validation		
	LS-OCMB status	Sex	Age	LS-OCMB status	Sex	Age	LS-OCMB status	Sex	Age
	P = 42	F = 24	34 (17–58)	P = 42	F = 24	34 (17–58)	P = 30	F = 18	33.4 (17–51)
		M = 18			M = 18			M = 12	
	N = 47	F = 39	37 (20–53)	N = 46	F = 38	37 (20–53)	N = 30	F = 24	36.9 (20–49)
		M = 8			M = 8			M = 6	

* Including patients in profiling cohort. Age—mean (range). LS-OCMB—lipid-specific oligoclonal IgM bands. P—positive. N—negative. F—females. M—males. There are 7 subjects (1 positive and 6 negative) in validation cohorts for which we do not have age data.

**Table 2:** ROC analysis results of the four candidate transcripts and different combinations. ROC—receiver operating characteristic.

Marker/Combination	Transcripts	Sample Size	AUC (%)	CI (%)
circRNA-1	circ_0000478	80 (43 LS-OCMB−; 37 LS-OCMB+)	65.9	53.9–77.9
circRNA-2	circ_0116639		66.4	54.2–78.5
Combi_circ	circ_0000478-circ_0116639		68.9	57.2–80.5
linearRNA-1	IRF5	55 (29 LS-OCMB−; 26 LS-OCMB+)	65.6	50.7–80.6
linearRNA-2	MTRNR2L8		67.0	52.4–81.7
Combi_linear	IRF5-MTRNR2L8		68.6	54.1–83.1
circRNA-1	circ_0000478	51 (26 LS-OCMB−; 25 LS-OCMB+)	65.1	49.8–80.4
circRNA-2	circ_0116639		66.2	50.6–81.7
linearRNA-1	IRF5		67.1	51.8–82.3
linearRNA-2	MTRNR2L8		69.0	54.1–83.9
CombiALL	circ_0000478-circ_0116639-IRF5-MTRNR2L8		69.8	55.3–84.4
combi1	circ_0000478-circ_0116639		66.3	51.1–81.6
combi2	IRF5-MTRNR2L8		70.3	55.6–85.0
combi3	circ_0000478-IRF5		68.3	52.8–83.8
combi4	circ_0000478-MTRNR2L8		68.6	53.7–83.6
combi5	circ_0116639-IRF5		69.8	55.0–84.7
combi6	circ_0116639-MTRNR2L8		67.8	52.8–82.9
combi7	circ_0000478-circ_0116639-IRF5		69.8	55.0–84.6
combi8	circ_0000478-circ_0116639-MTRNR2L8		68.2	53.3–83.0
combi9	circ_0000478-IRF5-MTRNR2L8		68.8	53.7–83.8
combi10	circ_0116639-IRF5-MTRNR2L8		68.8	53.8–83.7

## Supplementary Materials

Supplementary materials can be found at https://www.mdpi.com/2227-9059/8/12/540/s1. Table S1: A complete list of samples and the experiment in which each of them were included. Table S2: Assays and primers used in RT-qPCR experiments. Figure S1: Agarose gel electrophoresis image of PCR product and Sanger Sequencing results showing the back-spliced junction of circRNAs.
