# Supplementary figures and images for: Whole-Transcriptome Analysis in Peripheral Blood Mononuclear Cells from Patients with Lipid-Specific Oligoclonal IgM Band Characterization Reveals Two Circular RNAs and Two Linear RNAs as Biomarkers of Highly Active Disease
Source: Biomedicines. 2020 Nov 26;8(12):540. doi: 10.3390/biomedicines8120540 (PMC7759842; doi:10.3390/biomedicines8120540)

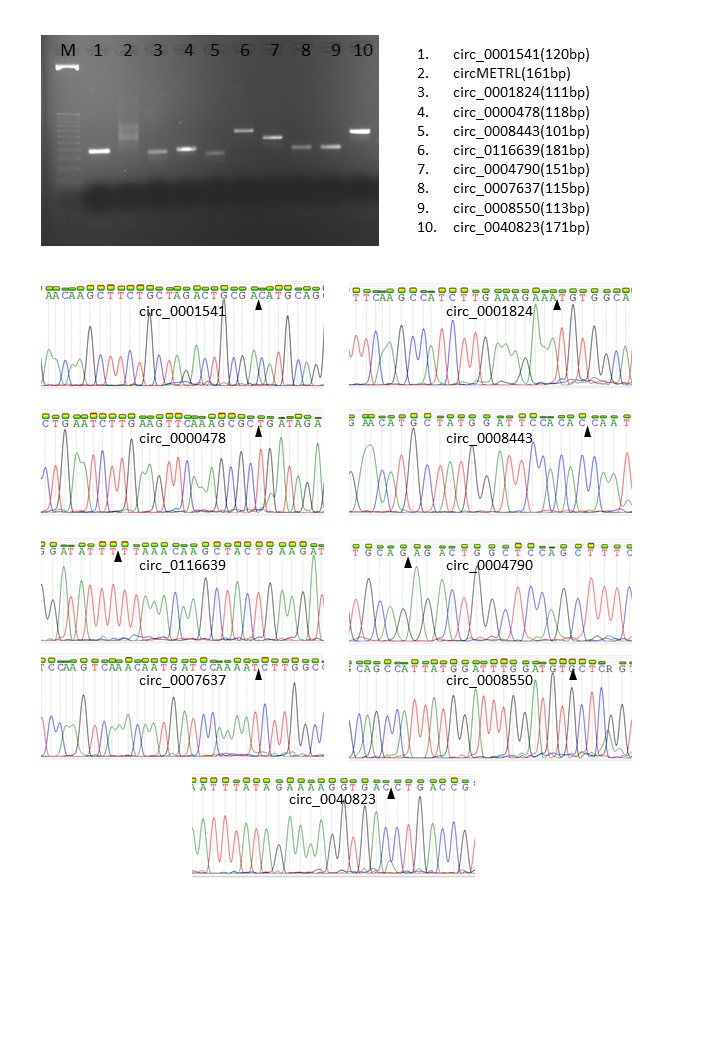

Supplement: Supplementary file 1 [file biomedicines-08-00540-s001.zip › biomedicines-995490 supplementary.v1/Figure S1.jpg]
